# Risk factors and mortality in patients with sepsis, septic and non septic acute kidney injury in ICU^a^

**DOI:** 10.1590/2175-8239-JBN-2018-0240

**Published:** 2019-09-16

**Authors:** Kellen Hyde Elias Pinheiro, Franciana Aguiar Azêdo, Kelsy Catherina Nema Areco, Sandra Maria Rodrigues Laranja

**Affiliations:** 1 Universidade Federal de São Paulo, Escola Paulista de Medicina, Departamento de Nefrologia, São Paulo, SP, Brazil.; 2 Hospital do Servidor Público Estadual de São Paulo, Francisco Morato de Oliveira, São Paulo, SP, Brazil.

**Keywords:** Acute Kidney Injury, Renal Insufficiency, Chronic, Sepsis, Nephrology, Water Balance, Mortality, Lesão Renal Aguda, Insuficiência Renal Crônica, Sepse, Nefrologia, Balanço Hídrico, Mortalidade

## Abstract

Acute kidney injury (AKI) has an incidence rate of 5-6% among intensive care unit (ICU) patients and sepsis is the most frequent etiology.

**Aims:**

To assess patients in the ICU that developed AKI, AKI on chronic kidney disease (CKD), and/or sepsis, and identify the risk factors and outcomes of these diseases.

**Methods:**

A prospective observational cohort quantitative study that included patients who stayed in the ICU > 48 hours and had not been on dialysis previously was carried out.

**Results:**

302 patients were included and divided into: no sepsis and no AKI (nsnAKI), sepsis alone (S), septic AKI (sAKI), non-septic AKI (nsAKI), septic AKI on CKD (sAKI/CKD), and non-septic AKI on CKD (nsAKI/CKD). It was observed that 94% of the patients developed some degree of AKI. Kidney Disease Improving Global Outcomes (KDIGO) stage 3 was predominant in the septic groups (*p* = 0.018). Nephrologist follow-up in the non-septic patients was only 23% vs. 54% in the septic groups (*p* < 0.001). Dialysis was performed in 8% of the non-septic and 37% of the septic groups (*p* < 0.001). Mechanical ventilation (MV) requirement was higher in the septic groups (*p* < 0.001). Mortality was 38 and 39% in the sAKI and sAKI/CKD groups vs 16% and 0% in the nsAKI and nsAKI/CKD groups, respectively (*p* < 0.001).

**Conclusions:**

Patients with sAKI and sAKI/CKD had worse prognosis than those with nsAKI and nsAKI/CKD. The nephrologist was not contacted in a large number of AKI cases, except for KDIGO stage 3, which directly influenced mortality rates. The urine output was considerably impaired, ICU stay was longer, use of MV and mortality were higher when kidney injury was combined with sepsis.

## Resumo

A Lesão Renal Aguda (LRA), cuja etiologia mais frequente é sepse, tem incidência de 5-6% na Unidade de Terapia Intensiva (UTI).

**Objetivo:**

Avaliar pacientes que permaneceram mais de 48 horas na UTI e desenvolveram LRA ou Doença Renal Crônica agudizada (DRCag) e/ou sepse; identificar fatores associados e causas que possam afetar a evolução desses pacientes.

**Método:**

Estudo prospectivo, observacional, coorte e quantitativo dos pacientes em UTI entre maio a dezembro de 2013 com sepse e LRA. Excluídos pacientes < 48 horas e/ou dialíticos prévios.

**Resultados:**

Dos 1156 pacientes admitidos, 302 foram incluídos e divididos em grupos: sem sepse e sem LRA (SSSLRA), apenas sepse (S), LRA séptica (LRAs), LRA não séptica (LRAns), DRCag séptica (DRCags), DRCag não séptica (DRCagns). Foi verificado que 94% apresentaram algum grau de lesão renal; Kidney Disease Improving Global Outcomes (KDIGO) 3 foi predominante nos grupos sépticos (*p* = 0.018); o nefrologista foi chamado apenas em 23% dos pacientes não sépticos vs. 54% dos sépticos (*p* < 0.001); houve necessidade de diálise em 8% dos não sépticos vs. 37% dos sépticos (*p* < 0.001); necessidade de Ventilação Mecânica (VM) em 61% da LRAns versus 90% na LRAs (*p* < 0.001). A mortalidade foi 38% e 39% na LRAs e DRCags vs. 16% e 0% na LRAns e DRCagns, respectivamente (*p* < 0.001).

**Conclusão:**

LRAs e DRCags têm pior prognóstico que a não séptica. O nefrologista ainda não é solicitado em grande parte dos casos com influência direta na mortalidade (*p* < 0.001), o débito urinário é consideravelmente prejudicado; o tempo de permanência na UTI, necessidade de VM e mortalidade são maiores quando há associação da sepse e LRA.

## Introduction

Acute kidney injury (AKI) is defined as a sudden reduction of renal function, increase of serum creatinine (sCr), and/or decrease of urine output (UO) and is a common complication in intensive care unit (ICU) patients^1^^,^ 2.

There is a strong association between prior chronic kidney disease (CKD) and AKI incidence during hospital stay; some authors describe prior CKD as a main risk factor for the development of AKI in the hospital^3^.

Sepsis is defined by the presence of infection associated with a systemic inflammatory response and has been the most important etiology for AKI in the ICU; this incidence can range from 11 to 70%^1^^,^ 4 ^,^ 5.

Therapeutic options that allow antibiotic therapy and maintenance of hemodynamic stability are still limited. The most important strategy is AKI prevention^6^.

Mortality in AKI is still extremely high and can affect 40-80% of ICU patients; the association between sepsis and AKI has a high mortality regardless of the primary diagnosis, whether sepsis or AKI, and the mortality increases significantly if there is need for renal replacement therapy (RRT)^1^^,^ 4 ^,^ 7 ^-^ 11.

Septic AKI is a great cause of mortality in ICU, it increases treatment costs, prolongs the length of stay in the hospital, worsens the prognosis of the patients, and increases the chance of CKD development^1^^,^ 4 ^,^ 5 ^,^ 12.

Information on septic AKI is still limited. Therefore, it is important to identify the profile of each hospital and the risk factors associated with the development of AKI or AKI on CKD (AKI/CKD). The aims of this research were to assess patients in the ICU that developed AKI, AKI on CKD, and/or sepsis and identify the risk factors and the outcomes of these diseases.

## Materials and methods

This prospective cohort observational quantitative study assessed all patients who stayed in the ICU for more than 48 hours at the State Public Hospital of São Paulo-HSPE/SP, a general tertiary and teaching hospital, from May to December 2013 and who developed AKI or AKI/CKD and/or sepsis.

Patients with an ICU stay < 48 hours and a history of dialysis-dependent CKD were excluded. A total of 1156 patients were admitted to the ICU during this period and finally 302 patients were included in the study.

This study was approved by the ethics committee of HSPE and signed informed consent was waived due to the observational nature of the study.

The population consisted of patients who stayed in the ICU for more than 48 hours and were diagnosed with AKI or AKI/CKD with or without sepsis and patients diagnosed with sepsis only or no sepsis and no AKI.

Sepsis was defined according to the International Guidelines for Management of Sepsis 2012^10^.

AKI was defined in accordance with the Kidney Disease Improving Global Outcomes (KDIGO) criteria as any of the following:

- Increase in sCr by ≥ 0.3 mg/dL within 48 hours;
- Or increase in sCr to ≥ 1.5 times baseline, which is known or presumed to had occurred within the prior 7 days;
- Or urine volume < 0.5 mL/kg/h for 6 hours.

KDIGO stages were defined as follows:

Stage 1, sCr 1.5 - 1.9 times baseline or ≥ 0.3 mg/dL increase or urine output < 0.5 mL/kg/h for 6 - 12 hours;
Stage 2, sCr 2.0 - 2.9 times baseline or urine output < 0.5 ml/kg/h for ≥ 12 hours;
Stage 3, sCr 3.0 times increase baseline or ≥ 4.0 mg/dL or urine output < 0.3 ml/kg/h for ≥ 24 hours or anuria for ≥12 hours or initiation of renal replacement therapy^11^.

Creatinine used for AKI diagnosis was the first sCr value measured during ICU stay or the previous sCr value before hospital admission registered in medical records.

Patients with pre-existing renal dysfunctions and a glomerular filtration rate < 60 mL/min were classified as having CKD^13^.

AKI/CKD was defined as worsening of renal function according to KDIGO^13^.

Daily assessments were performed to ensure that patients met the criteria for diagnosis of kidney injury and sepsis.

The patients were classified into six groups:

1. nsnAKI, no sepsis and no AKI.
2. S, sepsis without AKI or CKD.
3. sAKI, septic AKI.
4. nsAKI, nonseptic AKI.
5. sAKI/CKD, septic AKI on CKD.
6. nsAKI/CKD, nonseptic AKI on CKD.

**Figure 1:**
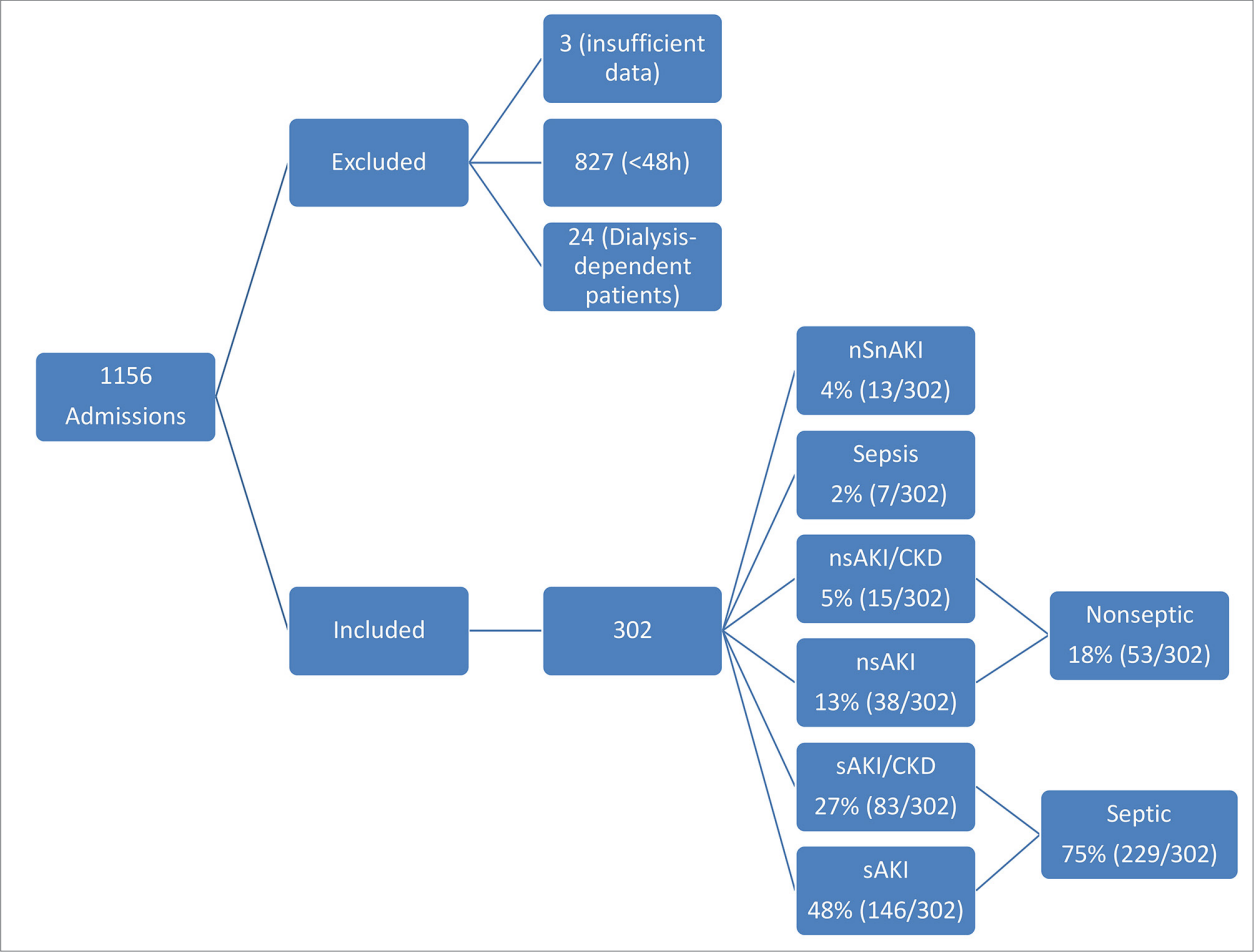
Sample Organization Diagram.

In some statistical analyses, the nsAKI and nsAKI/CKD groups were merged with the nonseptic group, whereas the sAKI and sAKI/CKD groups were merged with the septic group (Figure 1).

Data was collected from patients daily ICU history and evolution charts. Age, sex, race, weight, personal history, type of admission, and hospital stay were collected once. Minimum and maximum changes of vital signs, laboratory results, use of mechanical ventilation (MV), 6-, 12- and 24-h urine output, amount of fluid administered in 24 h, 24-h fluid balance, nephrologist follow-up, need for dialysis and its mode, and drugs and antibiotics were collected on a daily basis. Mortality was defined as death that occurred during ICU stay.

Data from the six groups underwent descriptive analysis: categorical variables were described by absolute (N) and relative (%) frequencies, and numerical variables were described by measures of central tendency (mean and median) and variability (interquartile range). A bivariate analysis was performed to compare the groups.

Association tests were performed to compare the groups regarding the numerical variables. A t-test was used for variables with normal distribution, and Kruskal-Wallis and Mann-Whitney tests were used for variables with non-normal distribution. For comparison of frequencies, the chi-square test and Fisher’s test were used. A *p* < 0.05 indicated significant associations or differences. The SPSS 13.0 software was used for statistical analysis.

A chi-square test was performed with linear association between sepsis and KDIGO classification variables.

Two multivariate analysis were performed with the following explicative variables or associated factors: age, sex, type of admission, ICU stay, 24-h fluid balance, nephrologist follow-up, and mechanical ventilation. One model considered the development of AKI as the final and the second multivariable analysis included death as final event.

## Results

We observed that 94% of the cases developed some degree of kidney injury (AKI or AKI/CKD), 77% had sepsis (sepsis, sAKI, and sAKI/CKD groups) mostly associated with kidney injury, 48% had sAKI, 27% had sAKI/CKD, while only 2% developed non-AKI associated sepsis. These results indicated a predominance of the combined diagnosis of sepsis and AKI (Figure 1).

Of the 302 patients included in the study, 54% were men and 89% were Caucasian; the median age was 71 years, 88% were emergency or urgent admissions, and the median ICU stay was 6 days (Table 1). The patients with sAKI (69 years) were younger than those with sAKI/CKD (76 years) (*p* < 0.001).

**Table 1:** Demographic data of the total casuistry

	n = 302
Male (%[n])	54 (162)
Caucasian (% [n])	89 (270)
Age (years [IQR])*	71 (62 - 79)
Urg./Emerg. Admission (% [n])	88 (265)
Pre-ICU Length of Stay (days [IQR])*	5 (1 - 18)
Length of Stay in ICU (days [IQR])*	6 (4 - 11)
Total Length of Stay (days [IQR])*	16 (8 - 27)
SAPS II [IQR]*	40 (32 - 50)

* Values in median.

The sAKI/CKD group had the lowest diuresis values in 24 hours, with an adjusted diuresis value of 0.58 mL/kg/hr (*p* = 0.013), followed by the nsAKI/CKD (0.83 mL/kg/hr), sAKI (0.98 mL/kg/hr), and nsAKI (0.96 mL/kg/h) groups. The nsnAKI (1.70 mL/kg/h) and S (1.81 mL/kg/h) groups had the highest urine output in 24 hours (*p* < 0.001) (Table 2).

**Table 2:** Demographic, Clinical and Laboratory Data of the Different Groups

	nsAKI	sAKI	nsAKI vs. sAKI	nsAKI/CKD	sAKI/CKD	nsAKI/CKD vs. sAKI/CKD	NSNAKI	S	All groups
	n = 38	n = 146	*p* value	n =15	n = 83	*p* value	n = 13	n = 7	*p* value
Male ( % [n])	42 % (16)	49 % (71)	0,473	73 % (11)	63% (52)	0,314	77% (10)	29% (2)	0,022
Caucasian (%[n])	89 % (34)	93 % (136)	0,446	93 % (14)	82% (68)	0,247	92% (12)	86% (6)	-
Age (years [IQR])*	73 (65 - 79)	69 (59 - 78)	0,243	77 (63 - 82)	76 (68 - 83)	0,657	64 (59 - 69)	70 (55-85)	0,001
Urg./Emerg. Admission (%[n])	74 % (28)	92 % (134)	0,002	87 % (13)	93% (77)	0,354	54% (7)	100% (7)	-
Pre-ICU Length of Stay (days[IQR])*	3 (1 - 13)	6 (1 - 20)	0,085	2 (1 - 6)	8 (2 - 21)	0,038	2 (1 -23)	11 (0 - 22)	0,163
Length of Stay in ICU (days[IQR])*	4 (3 - 6)	8 (5 - 12)	*p* < 0,001	4 (3 - 5)	8 (4 - 13)	*p* < 0,001	3 (3 - 4)	4 (3 - 8)	*p* < 0,001
Total Length of Stay (days[IQR])*	8 (5 - 17)	17 (10 - 31)	0,001	7 (4 - 11)	18 (11 - 31)	*p* < 0,001	5 (5 - 26)	14 (4 - 27)	*p* < 0,001
Urinary output in 24h (ml [IQR])*	1778 (1192 - 2488)	1602 (919 - 2281)	0,168	1329 (660 - 1475)	1338 (540 - 2017)	0,130	1750 (895 - 3191)	2583 (1675 - 3125)	0,013
Urinary output in 12h (ml [IQR])*	669 (526 - 1017)	620 (336 - 800)	0,909	358 (225 - 625)	472 (200 - 738)	0,503	867 (481 - 1131)	950 (733 - 1163)	0,001
Urinary output in 6h (ml [IQR])*	236 (161 - 324)	213 (100 - 313)	0,102	103 (90 - 269)	172 (67 - 293)	0,518	242 (126 - 517)	367 (350 - 413)	0,014
24-hour Fluid Balance (ml[IQR])*	561 (129 - 1135)	840 (298 - 1459)	0,950	1162 (463 - 1912)	990 (489 - 1609)	0,724	681 (-641 - 1221)	477 (-622 - 744)	0,020
24-hour Fluid Balance/Kg (ml[IQR])*	7,5 (2,8 - 13,8)	12 (3,8 - 20)	0,062	16 (5 - 29)	13 (6 - 21)	0,203	9 (-10,5 - 24,5)	7 (-9 - 10)	0,057
Diuresis in 6h (ml/kg/h [IQR])*	0,56 (0,35 - 0,77)	0,52 (0,24 - 0,73)	0,367	0,48 (0,27 - 0,69)	0,31 (0,10 - 0,54)	0,058	0,98 (0,76 - 1,46)	0,97 (0,95 - 1,19)	*p* < 0,001
Diuresis in 12h (ml/kg/h [IQR])*	0,74 (0,47 - 0,93)	0,72 (0,35 - 1,01)	0,367	0,67 (0,51 - 0,88)	0,46 (0,16 - 0,71)	0,012	1,37 (1,05 - 1,89)	1,32 (1,25 - 1,64)	*p* < 0,001
Diuresis in 24h (ml/kg/h [IQR])*	0,96 (0,65 - 1,27)	0,98 (0,52 - 1,29)	0,571	0,83 (0,70 - 1,10)	0,58 (0,25 - 0,96)	0,013	1,70 (1,29 - 2,45)	1,81 (1,66 - 1,99)	*p* < 0,001
Serum Creatinine (mg/dL [IQR])*	1,3 (0,9 - 2,3)	1,4 (0,9 - 2,2)	0,695	1,4 (0,8 - 2,4)	1,5 (1 - 2,3)	0,414	0,9 (0,7 - 1,1)	1 (0,5 - 2,1)	0,560
Urea (mg/dL [IQR])*	81 (53 - 109)	86 (54 - 123)	0,435	100 (76 - 130)	84 (54 - 125)	0,584	99 (40 - 117)	76 (26 - 85)	0,615
Nephrologist follow-up (%[n])*	13 % (5)	42 % (62)	0,001	47% (7)	75% (62)	0,029	-	-	-
Mechanical ventilation (%[n])	61 % (23)	90 % (131)	*p* < 0,001	67% (10)	88% (73)	0,051	62% (8)	57 % (4)	*p* < 0,001
Length of mechanical ventilation [IQR]*	1 (0 - 4)	6 (3 - 11)	*p* < 0,001	1 (0 - 3)	7 (3 - 13)	0,001	1 (0 - 2)	2 (0 - 4)	*p* < 0,001
SAPS II [IQR]*	35 (28 - 39)	42 (32 - 51)	*p* < 0,001	45 (39 - 49)	44 (37 - 51)	0,325	30 (26 - 34)	40 (27 - 61)	*p* < 0,001
Death (%[n])*	16 % (6)	38 % (56)	0,00875137	0	39% (32)	0,001	0	0	-

* Values in median Significant p < 0,05.

Decreased renal function was related with high 24-h fluid balance (FB) values, progressively greater accumulation in the S, nsAKI, sAKI, sAKI/CKD, and nsAKI/CKD groups, and a mean of 477 mL/24 h in the S group reaching 1162 mL/24 h in the nsAKI/CKD group (*p* = 0.020) (Table 2).

The sCr values did not show significant differences among groups (Table 2).

Emergency and urgent hospitalizations were significantly higher in the septic group (92%) compared to the non-septic group (77%) (*p* = 0.002)(Table 3). Additionally, the median ICU stay and total hospital stay were significantly greater in the septic groups and were double those in the nonseptic groups (*p* < 0.001) (Table 3). There was no difference in ICU stay between the sAKI and sAKI/CKD groups (Table 3).

**Table 3:** Comparison of Acute Kidney Injury vs. Chronic Kidney Disease and Septic vs. Nonseptic

	nsAKI	nsAKI/CKD	nsAKI vs. nsAKI/CKD	sAKI	sAKI/CKD	sAKI vs. sAKI/CKD	Nonseptic	Septic	Nonseptic vs. Septic
	n = 38	n =15	*p* value	n =146	n = 83	*p* value	n = 53	n = 229	*p* value
Male ( % [n])	42 % (16)	73 % (11)	0,041	49 % (71)	63 % (52)	0,041	51 % (27)	54 % (123)	0,716
Caucasian ( % [n])	89 % (34)	93 % (14)	0,561	93 % (136)	82 % (68)	0,009	91 % (48)	89% (204)	0,752
Age (years [IQR])*	73 (65 - 79)	77 (63 - 82)	0,210	69 (59 - 78)	76 (68 - 83)	*p* < 0,001	74 (65 - 81)	72 (62 - 79)	0,367
Urg./Emerg. Admission (%[n])	74% (28)	87 % (13)	0,309	92 % (134)	93% (77)	0,789	77 % (41)	92 % (211)	0,002
Pre-ICU Length of Stay (days [IQR])*	3 (1 - 13)	2 (1 - 6)	0,690	6 (1 - 20)	8 (2 - 21)	0,494	2 (1 - 11)	7 (1 - 20)	0,009
Length of Stay in ICU (days [IQR])*	4 (3 - 6)	4 (3 - 5)	0,523	8 (5 - 12)	8 (4 - 13)	0,897	4 (3 - 5)	8 (4 - 12)	*p* < 0,001
Total Length of Stay (days [IQR])*	8 (5 - 17)	7 (4 - 11)	0,368	17 (10 - 31)	18 (11 - 31)	0,386	8 (5 - 15)	18 (10 - 31)	*p* < 0,001
CR Change (↑) (% [n])	5 % (2)	7 % (1)	0,640	6 % (9)	1 % (1)	0,077	6 % (3)	4 % (10)	0,686
Urine output change (↓)(% [n])	47 % (18)	20 % (3)	0,067	23 % (34)	13 % (11)	0,066	40 % (21)	20 % (45)	0,002
Change of two parameters (% [n])	47 % (18)	73 % (11)	0,087	71 % (103)	86 % (71)	0,011	55 % (29)	76 % (174)	0,002
KDIGO 1 (% [n])	16 % (6)	13 % (2)	0,822	15 % (22)	12 % (10)	0,526	15 % (8)	14 % (32)	0,833
KDIGO 2 (% [n])	61 % (23)	40 % (6)	0,176	36 % (52)	28 % (23)	0,220	55 % (29)	33 % (75)	0,003
KDIGO 3 (% [n])	24 % (9)	47 % (7)	0,101	49 % (72)	60 % (50)	0,111	30 % (16)	53 % (122)	0,002
Nephrologist Follow-up (% [n])	13 % (5)	47 % (7)	0,009	4 2% (62)	75 % (62)	*p* < 0,001	23 % (12)	54 % (124)	*p* < 0,001
Dialysis (% [n])	0	27 % (4)	0,005	32 % (47)	45 % (37)	0,062	8 % (4)	37 % (84)	*p* < 0,001
SAPS II [IQR]*	35 (28 - 39)	45 (39 - 49)	0,002	42 (32 - 51)	44 (37 - 51)	0,275	37 (29 - 43)	43 (34 - 51)	*p* < 0,001
Death (% [n])	16 % (6)	0	0,120	38 % (56)	39 % (32)	0,976	11 % (6)	38 % (88)	*p* < 0,001

* Values in median Significant p < 0,05.

Patients with sAKI and sAKI/CKD required significantly more MV compared to the septic patients without kidney injury (S) group (respectively, 90%, 88%, and 57%, *p* < 0.001). The duration of MV was also greater in septic groups and was 2-fold that of the other groups (*p* < 0.001) (Table 2).

The comparison of the severity score (Simplified Acute Physiology Score II, SAPS II), among patients with sAKI (42) and nsAKI (35) showed a significant difference (*p* < 0.001), but there was no significant difference between sAKI/CKD (44) and nsAKI/CKD (45) (*p* = 0.325) (Table 2). Mortality was greater in the sAKI group (38%) and sAKI/CKD group (39%). This indicated that the combination of renal injury and sepsis increases ICU mortality. It is noteworthy that there was no death in the nsAKI/CKD group (Tables 2 and 3).

Patients with AKI were followed up by the nephrologist significantly fewer times than the AKI/CKD patients (42% in the sAKI group vs 75% in sAKI/CKD group (*p* = 0.009)) (Table 3). The septic group also required more RRT, especially patients with sAKI/CKD (45%). This group also presented a higher mortality (*p* < 0.001) (Table 3).

**Figure 2:**
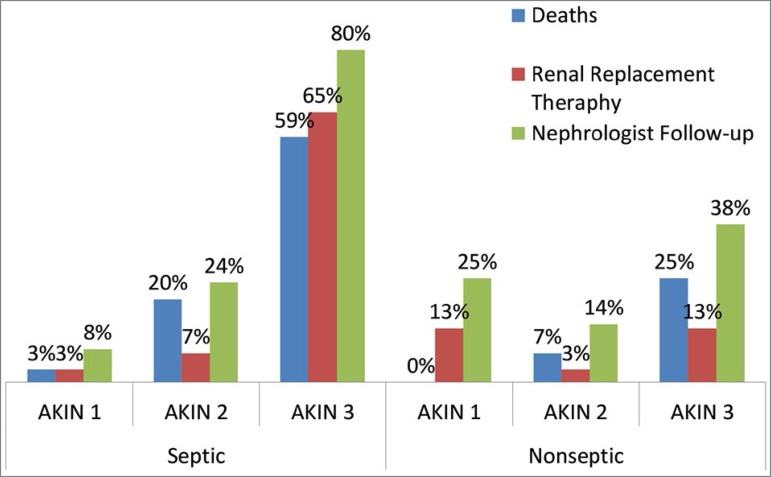
Nephrologist Follow-up.

Most patients followed by the nephrologist were those diagnosed with KDIGO 3 in both the septic and nonseptic groups (80% and 38%, respectively) (*p* < 0.001). Septic patients in KDIGO 1 had only 8% of nephrologist follow-up vs 80% of those classified in KDIGO 3. The need of RRT and mortality were significantly greater in septic KDIGO 3 patients (65 and 59%, respectively) (*p* < 0.001) (Figure 2).

The bivariate analysis showed a linear trend between the severity of AKI and the incidence of sepsis, showing greater involvement of KDIGO 3 in septic patients (Table 4).

**Table 4:** C hi-square test of KDIGO and sepsis

KDIGO vs. Sepse				
		Sepse		Total
		No	Yes	
KDIGO	1	8	32	40
	2	29	75	104
	3	16	122	138
Total		53	229	282

Pearson's Chi-square *p* = 0,006

Linear by Linear Association *p* = 0,026.

On multivariate analysis, type of admission, ICU stay, MV, and nephrologist follow-up were the determining factors for developing sAKI (Table 5).

**Table 5:** Multivariate analysis Table 5 of sAKI and death associated

sAKI Associated Factors	*p*	OR (I.C. 95%)
Nephrologist Follow-up	0,003	
no		3,112 (1,462 - 6,622)
yes		1,000
Mechanical Ventilation	0,027	
yes		2,417 (1,107 - 5,277)
no		1,000
Length of Stay in the ICU	*p* < 0,001	1,336 (1,155 - 1,545)
Type of admission	0,061	
urg/emer		2,377 (0,961 - 5,881)
elective		1,000
Constant	*p* < 0,001	0,118
Death Associated Factors	*p*	OR (I.C. 95%)
Nephrologist Follow-up	*p* < 0,001	
no		5,361(3,013 - 9,539)
yes		1,000
Mechanical Ventilation	0,006	
yes		4,800 (1,580 - 14,588)
no		1,000
24-hour Fluid Balance	0,045	1,000 (1,000 - 1,001)
Length of Stay in the ICU	0,073	1,039 (0,996 - 1,083)
Constant	*p* < 0,001	0,024

The ICU stay of sAKI and sAKI/CKD was twice that of other patients. Each day of stay in the ICU increased in 33% the probability of developing sAKI (*p* < 0.001) (Table 5). The need of MV also increased the probability of developing sAKI (*p* = 0.027) and the absence of nephrologist assistance increased by 211% this probability (*p* = 0.003) (Table 5).

On multivariate analysis with death as the final event, the significantly associated factors were absence of nephrologist follow-up, MV, 24-h FB, and ICU stay (Table 5).

## Discussion

AKI has a multifactorial etiology and is common in the ICU environment. It must be identified early and readily treated. In addition, aggravating factors must be identified and modified as soon as possible. Studies have suggested that even lighter degrees of AKI contribute to the development of CKD and increase in mortality^2^^-^ 9 ^,^ 11.

In this prospective study, 94% of ICU-admitted patients who stayed for a period longer than 48 h developed some degree of kidney injury; 75% of these patients had kidney injury and sepsis. This incidence is higher than that found in the literature in recent studies. Hoste (2015) shows an incidence of 54% on the 1st and 2nd day of ICU stay, and longer ICU stays can expose patients to a higher risk of AKI^1^^,^ 4 ^,^ 5 ^,^ 8.

Patients with AKI/CKD were older compared to the total sample (77 and 71 years, respectively) (Table 1), whereas the median age in the sAKI group was 69 years (Table 2), similar to the BEST Kidney study; age was not a factor associated with mortality^1^.

In all groups, emergency and urgent hospitalizations prevailed in the sAKI (92%) and sAKI/CKD (93%) groups. Bagshaw et al. (2008) showed similar results^8^.

ICU stay was significantly longer when kidney injury was associated with sepsis; likewise, the total hospital stay showed that septic kidney injury increased ICU stay, reaching twice the admission period compared to that with non-septic kidney injury, showing that the increase in ICU stay is associated with sepsis^7^^,^ 14.

There was a high incidence of hypertension (H), diabetes mellitus (DM), heart failure (HF), and neoplasms, particularly in the CKD group compared to the other groups. Notably, DM was higher, which can be explained by the more advanced age of these patients. Neoplasms required attention in all studied groups as it was the fourth most frequently concomitant disease, with the exception of the sAKI/CKD group (electronic Annex 1).

In our study, there was a progressive decrease of UO in the nsAKI, AKI, and AKI/CKD groups, and a further decrease in the septic groups. UO was considerably more impaired when there was an association between kidney injury and sepsis. Simultaneously, there was a progressive increase in FB in the same groups reaching more than 1L in the sAKI/CKD group. Studies have shown that a positive FB can worsen the condition of critical patients and underestimate diagnosis due to sCr dilution, leading to increased mortality^2^^,^ 15.

The patients’ weight-adjusted 24-h FB was 7.5 mL/kg in the nsAKI group and 12 mL/kg in the sAKI group (Table 2). Although this difference was not statistically significantly, it is important in clinical practice^15^.

It has been difficult to determine whether fluid overload is a worsening marker for sAKI or the cause of mortality increase^15^. Studies have shown that volume resuscitation, outside the therapeutic window, is useless and may be harmful^16^^,^ 17.

Except for the nsAKI group, there was a predominance of the KDIGO 3 level in other groups.

The nephrologist was consulted more often in the follow-up of septic patients (sAKI, 42%; sAKI/CKD, 75%); however, this rate is still low if we consider that the diagnostic classification of AKI is familiar to the hospital’s intensive care physicians. Knowing that the changes of sCr and UO are not the best diagnostic markers for AKI, we can consider that KDIGO stage 1 is the ideal stage to consult the nephrologist. However, this often occurred late, when the patient was in KDIGO stage 3, and in less than half of the patients, with the exception of the sAKI/CKD group (nsAKI, 13%; sAKI, 42%; nsAKI/CKD, 27%; sAKI/CKD, 75%). Although the follow-up was greater in the septic group, it was still only 54%.

Patients with CKD were also more likely to be followed up by the nephrologist, suggesting that the awareness of kidney disease brings attention to the need of follow-up; however, more than half of the AKI cases were still being managed by the intensivist exclusively. The absence of nephrologist follow-up was the main risk factor associated with increased mortality (OR = 5.3). Thus, the habit of requesting the nephrologist’s evaluation in the early stages of AKI still needs to be reinforced^1^^,^ 2 ^,^ 4.

Patients with sepsis required more RRT (37% of septic AKI vs 8% in non-septic AKI), used more frequently nephrotoxic drugs and combined antibiotics (electronic Annex 2), had a higher need for MV, presented with higher mortality, showing once again that sepsis and kidney injury combined lead to a worse prognosis^4^^,^ 18. Critical patients have a high incidence of infection and antimicrobial therapy can be a cause of AKI; likewise, AKI can facilitate infection development, making it difficult to understand the cause and effect relationship.

The use of nephrotoxic drugs averages a 19% contributing factor to AKI in critical patients. These can be identified and sometimes replaced after the nephrologist follow-up^19^. For example, hydroxyethylamide (Voluven^®^) was used by 5% of the patients in the nsAKI, sAKI, and sAKI/CKD groups during this study despite it being widely contraindicated in sepsis and renal failure cases, which could be prevented with the nephrologist follow-up^19^.

In this study, high rates of SAPS II were found in the sAKI/CKD (44), sAKI (42) groups, as well as in the nsAKI/CKD (45) group. Although mortality was high in the septic groups, in the nsAKI/CKD, which had high SAPS II index, had no death. Thus, CKD itself was not the decisive factor for increasing severity or mortality, but rather the combination of kidney injury with sepsis.

This can be observed also in the comparison of septic vs non-septic patients, where the non-septic group had lower SAPS II and mortality rates. Diverse studies point out that AKI is an independent risk factor for mortality when associated with sepsis. It is worth emphasizing that mortality was progressively higher with higher KDIGO stages (KDIGO 3) and in patients with sAKI (Figure 2)^2^^,^ 4 ^,^ 6 ^,^ 7 ^,^ 20.

The main factors associated with the risk of developing sAKI were urgent or emergency admission, ICU stay, lack of nephrologist follow-up, and MV need. On multivariate analysis to evaluate mortality, the more strongly associated factors were KDIGO stage 3 AKI, MV need, and absence of nephrologist follow-up, with highly significant values and OR. MV use and 24-h fluid balance increase also showed a strong association with mortality. ICU stay, although quite different among groups, was not a determining factor (*p* = 0.073). It is worth discussing whether these factors lead to the development of LRAs or whether LRAs are responsible for increasing these parameters^21^.

## Conclusion

We conclude that sepsis was the main factor associated with AKI (75%) in this ICU study. AKI associated with sepsis had the worst outcomes (38% mortality) compared to non-septic AKI (16% mortality). Sepsis also worsened the prognosis of patients with AKI/CKD (39% mortality) compared to non-septic AKI/CKD (no death). Septic patients with no AKI had a more preserved UO compared to that in all groups with AKI or AKI/CKD. The need for mechanical ventilation was higher in the sAKI (90%) and sAKI/CKD (88%) groups compared to the nsAKI (61%) or nsAKI/CKD (67%) groups, as well as the duration of mechanical ventilation, (nsAKI, 1 day and nsAKI/CKD, 1 day vs. sAKI, 6 days and sAKI/CKD, 7 days). The nephrologist was not consulted in the first stages of AKI, showing that the need for consulting the nephrologist in the early stages of AKI must be highlighted. Patients with AKI/CKD were followed-up by nephrologists more often probably because of the previous knowledge of CKD.

## Supplementary material

The following online material is available for this article:

Annex 1.

Annex 2.
