# Supplementary material for: Risk factors and mortality in patients with sepsis, septic and non septic acute kidney injury in ICU
Source: J Bras Nefrol. 2019 Sep 16;41(4):462–71. doi: 10.1590/2175-8239-JBN-2018-0240 (PMC6979581; doi:10.1590/2175-8239-JBN-2018-0240)
Supplement: Supplementary file 1 [file 2175-8239-jbn-2018-0240-suppl1.pdf]

Supplementary Material to "Risk factors and mortality in patients with sepsis, septic and non septic acute kidney injury in ICU"

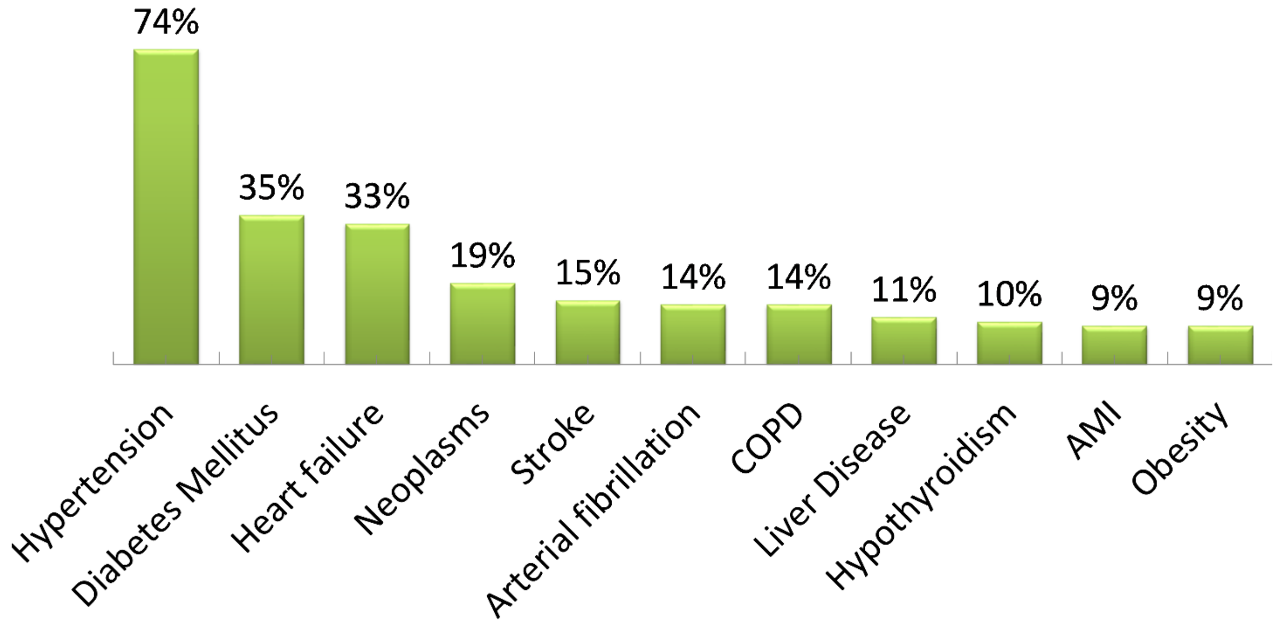

Annex 1 - Associated Comorbidities in Total Casuistry.
