# Supplementary material for: Risk factors and mortality in patients with sepsis, septic and non septic acute kidney injury in ICU
Source: J Bras Nefrol. 2019 Sep 16;41(4):462–71. doi: 10.1590/2175-8239-JBN-2018-0240 (PMC6979581; doi:10.1590/2175-8239-JBN-2018-0240)
Supplement: Supplementary file 2 [file 2175-8239-jbn-2018-0240-suppl2.pdf]

Supplementary Material to "Risk factors and mortality in patients with sepsis, septic and non septic acute kidney injury in ICU"

Annex 2. Drugs used.

	LRAns N=38	LRAs N=146	DRCagns N=15	DRCags N=83	P
**Diuretic**	47%	64%	60%	64%	0,113
**1 vasoactive drug**	40%	53%	33%	54%	0,225
**2 ou + associated vasoactive drug**	37%	26%	40%	34%	0,369
**IECA**	29%	20%	20%	12%	0,160
**1 nephrotoxic antibiotic**	16%	29%	-	40%	0,003
**2 ou + associated nephrotoxic antibiotic**	-	28%	-	22%	< 0,001
**Hydroxyethylamido**	5%	5%	-	5%	-
**Antiviral Nephrotoxic**	-	1%	-	7%	-
**Antifungal Nephrotoxic**	-	1%	-	4%	-

Antibiotics = vancomycin, amikacin, polymyxin, gentamicin and rifampicin

IECA = inhibitors of angiotensin converting enzyme
